# A Comprehensive Assessment of Lymphatic Filariasis in Sri Lanka Six Years after Cessation of Mass Drug Administration

**DOI:** 10.1371/journal.pntd.0003281

**Published:** 2014-11-13

**Authors:** Ramakrishna U. Rao, Kumara C. Nagodavithana, Sandhya D. Samarasekera, Asha D. Wijegunawardana, Welmillage D. Y. Premakumara, Samudrika N. Perera, Sunil Settinayake, J. Phillip Miller, Gary J. Weil

**Affiliations:** 1 Department of Internal Medicine, Infectious Diseases Division, Washington University School of Medicine, St. Louis, Missouri, United States of America; 2 Anti Filariasis Campaign, Sri Lanka Ministry of Health, Colombo, Sri Lanka; 3 Division of Biostatistics, Washington University School of Medicine, St. Louis, Missouri, United States of America; Institute of Medical Microbiology, Immunology and Parasitology, Germany

## Abstract

**Background:**

The Sri Lankan Anti-Filariasis Campaign conducted 5 rounds of mass drug administration (MDA) with diethycarbamazine plus albendazole between 2002 and 2006. We now report results of a comprehensive surveillance program that assessed the lymphatic filariasis (LF) situation in Sri Lanka 6 years after cessation of MDA.

**Methodology and Principal Findings:**

Transmission assessment surveys (TAS) were performed per WHO guidelines in primary school children in 11 evaluation units (EUs) in all 8 formerly endemic districts. All EUs easily satisfied WHO criteria for stopping MDA. Comprehensive surveillance was performed in 19 Public Health Inspector (PHI) areas (subdistrict health administrative units). The surveillance package included cross-sectional community surveys for microfilaremia (Mf) and circulating filarial antigenemia (CFA), school surveys for CFA and anti-filarial antibodies, and collection of *Culex* mosquitoes with gravid traps for detection of filarial DNA (molecular xenomonitoring, MX). Provisional target rates for interruption of LF transmission were community CFA <2%, antibody in school children <2%, and filarial DNA in mosquitoes <0.25%. Community Mf and CFA prevalence rates ranged from 0–0.9% and 0–3.4%, respectively. Infection rates were significantly higher in males and lower in people who denied prior treatment. Antibody rates in school children exceeded 2% in 10 study sites; the area that had the highest community and school CFA rates also had the highest school antibody rate (6.9%). Filarial DNA rates in mosquitoes exceeded 0.25% in 10 PHI areas.

**Conclusions:**

Comprehensive surveillance is feasible for some national filariasis elimination programs. Low-level persistence of LF was present in all study sites; several sites failed to meet provisional endpoint criteria for LF elimination, and follow-up testing will be needed in these areas. TAS was not sensitive for detecting low-level persistence of filariasis in Sri Lanka. We recommend use of antibody and MX testing as tools to complement TAS for post-MDA surveillance.

## Author Summary

Lymphatic Filariasis (LF, also known as “elephantiasis”) is a disabling and deforming disease that is caused by parasitic worms that are transmitted by mosquitoes. The Sri Lankan Anti-Filariasis Campaign provided five annual rounds of mass drug administration (MDA) with diethylcarbamazine and albendazole between 2002 and 2006 in all endemic areas (districts or implementation units), and this reduced infection rates to very low levels in all sentinel and spot check sites. Transmission Assessment Surveys (TAS, surveys for filarial antigenemia in primary school children) performed in 2012–2013 (about 6 years after the last round of MDA) showed that all 11 evaluation units in formerly endemic areas easily satisfied a key World Health Organization target for LF elimination programs. More comprehensive surveillance was performed with other tests to assess LF parameters in 19 study sites in the same eight districts. We detected evidence of persistent LF in all districts and evidence of ongoing transmission in several areas. Exposure monitoring (screening for anti-filarial antibodies in primary school children) and molecular xenomonitoring (detecting filarial DNA in mosquito vectors) were much more sensitive than TAS for detecting low level persistence of filariasis in Sri Lanka. These methods are complementary to TAS, and they are feasible for use by some national filariasis elimination programs. Results from this study suggest that TAS alone may not be sufficient for assessing the success of filariasis elimination programs.

## Introduction

Lymphatic filariasis (LF, caused by the mosquito borne filarial nematodes *Wuchereria bancrofti*, *Brugia malayi*, and *B. timori*), is a major public-health problem in many tropical and subtropical countries. The latest summary from the World Health Organization (WHO) reported that 56 of 73 endemic countries have implemented mass drug administration (MDA) with a combination of two drugs (albendazole with either ivermectin or diethycarbamazine), and 33 countries have completed 5 or more rounds of MDA in some implementation units [1]. With more than 4.4 billion doses of treatment distributed between 2000 and 2012, the Global Programme to Eliminate Lymphatic Filariasis (GPELF) is easily the largest public health intervention to date based on MDA.

Bancroftian filariasis was highly endemic in Sri Lanka in the past [2]–[4]. The Sri Lankan Ministry of Health' Anti Filariasis Campaign (AFC) used a variety of methods to reduce filarial infection rates to low levels by 1999 [5], [6]. Sri Lanka was one of the first countries to initiate a LF elimination program based on GPELF guidelines [7]. The AFC provided annual MDA with diethylcarbamazine alone for three years starting in 1999. This was followed by five annual rounds of MDA with albendazole plus diethylcarbamazine in all 8 endemic districts (implementation units, IU) between 2002 and 2006. Various types of surveillance have been conducted by AFC and other groups since the MDA program ended in 2006 [8]–[12]. Post-MDA surveillance results (based on detection of microfilariae or Mf in human blood by microscopy) have consistently shown Mf rates much lower than the target value of 1% in all endemic areas [13]. The AFC also conducted school-based surveys for filarial antigenemia in 2008 according to WHO guidelines active at that time. Approximately 600 children were tested for circulating filarial antigenemia (CFA) in 30 schools in each of the 8 endemic districts, and no positive tests were observed (unpublished data, Sri Lanka Ministry of Health). WHO guidelines emphasize that LF elimination programs should provide care for people with acute and chronic clinical manifestations of filariasis [7], and the AFC has an excellent network of clinics that is devoted to this activity [13].

The present study represents a significant expansion of earlier post-MDA surveillance activities in Sri Lanka. Transmission assessment surveys (TAS) were performed according to current WHO guidelines [14], [15] for sampling primary school children to detect filarial antigenemia in each district. While TAS results may be useful for deciding whether MDA can be stopped, TAS cannot guarantee that LF transmission has been interrupted in evaluation units (EUs), which are typically districts with populations that may exceed 1 million. Therefore we conducted more intensive surveillance activities in smaller areas (Public Health Inspector “PHI” areas) that were considered to be at high risk for persistent filariasis to complement the TAS program.

Provisional targets have been proposed for documenting the interruption of filariasis transmission based on studies of the effects of MDA in Egypt, which also has LF transmitted by *Culex* mosquitoes [16]. Targets proposed for treated populations after at least five years of effective MDA were <2% for filarial antigenemia in communities (which corresponds to a MF prevalence rate of <0.5%), <2% for antibody to the recombinant filarial antigen Bm14 in first grade primary school children, and <0.25% for parasite DNA rates in mosquitoes as assessed by molecular xenodiagnosis (MX). The present study provided an opportunity to gain further experience with these parameters in the post-MDA setting.

Thus, the first aim of this study was to test the hypothesis that LF has been eliminated in Sri Lanka some 6 years after the completion of its national MDA program. The second aim was to assess the relative value of different methods for detecting low level persistence of filariasis after MDA.

## Methods

### Comprehensive surveillance surveys of Public Health Inspector (PHI) areas

Comprehensive surveillance activities in this project used Public Health Inspector (PHI) areas as sentinel sites. PHIs are sub-district health administration units that are comprised of smaller Public Health Midwife (PHM) areas. PHI's typically have populations in the range of 10,000–30,000 people, but they are larger in the country's capital city of Colombo which does not belong to a district. Post-MDA comprehensive surveillance studies were performed in at least two PHIs in each of the 8 LF-endemic districts in Sri Lanka plus two sites in Colombo town. The mean area of these PHIs was 6.3 km^2^ (range 0.6 km^2^–24.5 km^2^). Most PHIs selected for this study were considered to be at increased risk for persistent filariasis based on high infection rates prior to MDA or based on results of microfilaremia surveys conducted after 2006.

### Field procedures for community surveys and school surveys in Public Health Inspector (PHI) areas

Field teams for collection of demographic information and blood specimens consisted of a medical officer, a Public Health Inspector, a phlebotomist, and one or two assistants. Blood samples were collected during the day. Sterile, single use, contact activated BD-microtainer lancets (Fisher Scientific, Pittsburgh, PA) were used for blood collection in community and school surveys. Approximately 300 to 400 µl of blood was collected by finger prick from each study subject into an EDTA coated blood collection vial (Fisher Scientific). Barcode stickers were used to link specimens to data records. Samples were transported to the AFC headquarters laboratory in Colombo in coolers. Plasma was separated from blood samples from school children and stored at −80 C for later antibody testing.

### Community filariasis surveys in PHI areas

A pilot study was performed in Peliyagodawatta in Gampaha district in 2008 as a training exercise and to test the feasibility of comprehensive LF surveillance in Sri Lanka using methods pioneered in Egypt. This semi-urban area (with a population of about 10,560 in an area of 1.59 km^2^) was resurveyed in 2011. All other PHIs were only studied once.

The community surveys used a systematic sampling scheme to sample all areas in each PHM within the PHI being studied. The AFC obtained census lists with the numbers of houses in each PHM and PHI along with maps showing the PHMs within PHIs. The number of houses/households needed for each community survey (125) was divided by the number of PHMs in the PHI to get the number of houses to be sampled in each PHM. That number was divided by 4 to get the number of houses to be sampled per quadrant in each PHM. The central house in the quadrant was sampled, and other houses were selected by moving in the 4 cardinal directions from the central house. The sampling interval for houses was calculated by dividing the total number of houses in the PHM quadrant by the number of houses that were to be sampled in that quadrant. For instance, if there were 60 houses in a quadrant and 10 houses were to be sampled, the sampling interval was 6. If a selected house could not be sampled because of absence or refusal, field teams sampled the next house. Community surveys sampled people who were at least 10 years of age, and a maximum of 4 subjects were enrolled per house.

### School-based surveys for antifilarial antibodies and filarial antigenemia

Finger prick blood was collected from children in grades 1 and 2 in primary schools that served children in the study PHIs; approximately 350 school blood samples were collected per PHI. Blood was tested for filarial antigenemia by card test, and plasma was stored for later antibody testing.

### Collection of mosquitoes for filarial DNA detection

Mosquitoes were collected with gravid traps (Model 1712, John W. Hock Company, Gainesville, FL) using liquid bait. The liquid bait was prepared 5–6 days prior to use containing yeast, milk powder and dry straw in water [17]. In some PHI areas cow dung was added to the liquid bait to attract mosquitoes.

Gravid traps were placed adjacent to houses for one to four days; mosquitoes were collected in the morning and traps were replaced in the evening. Traps were placed in shaded, quiet areas near natural breeding sites. Traps were placed in all 4 quadrants of each PHM to ensure sampling from all areas in each PHI.

In the Peliyagodawatta pilot study in 2008, 4835 mosquitoes were collected from 20 trap sites, and the number of pools collected from each trap ranged from 1–10 pools of mosquitoes (range 5–20 mosquitoes per pool). In all subsequent surveys, 4 pools of twenty mosquitoes were collected from each of 50 trapping sites per PHI. Trapped mosquitoes were collected, sorted, dried at 95°C for 1 hr. and placed in tubes for later testing (20 mosquitoes/pool). The tubes were labeled with barcode stickers and transferred to the AFC headquarters laboratory for DNA isolation and qPCR testing.

### Laboratory testing of samples from PHI surveys

Washington University personnel trained staff in the central AFC laboratories on standard operating procedures for Mf detection by microscopy, antibody and antigen testing, DNA isolation from mosquitoes, and detection of filarial DNA by qPCR. All samples were tested in AFC laboratories in Colombo.

### Blood tests for filarial infection or exposure to filarial parasites

Circulating filarial antigenemia (CFA) was detected with a simple card test (BinaxNOW Filariasis, Alere Inc., Scarborough, ME) [16], [18].

IgG_4_ antibodies to recombinant filarial antigen Bm-14 in human plasma were detected by microplate ELISA (Filariasis CELISA, Cellabs Pty Ltd, Brookvale, NSW, Australia) as previously described [19]. Previous studies have shown that this kit is sensitive and specific for infection and/or heavy exposure to filarial parasites. Plasma ELISAs were performed with a single well per sample, and all positive and borderline tests were retested on a different day. Samples that produced an OD value >0.35 in two assays performed on different days were considered to be positive for antibody to Bm14.

Microfilaria (Mf) testing was performed for people with positive filarial antigen tests (in community household surveys, school surveys, and TAS) with three-line blood smears (60 µl total volume of night blood tested).

### Detection of filarial DNA in mosquitoes

Mosquitoes were sorted by experienced technicians. Blood fed, gravid, and semi-gravid *Culex quinquefaciatus* mosquitoes were identified by morphology and sorted into 4 pools of 20 mosquitoes per collection site. Two hundred and seventy-seven pools of mosquitoes (mean pool size of 17) were collected and tested from Peliyagodawatta in the pilot study that was performed in 2008. Approximately 200 pools were tested from each PHI area in later surveys. *W. bancrofti* DNA was detected in mosquito pools by qPCR as previously described [16], [20]. DNA isolation and PCR analysis for samples from the 2008 pilot study were performed by AFC personnel together with Washington University technicians in St. Louis. All subsequent PCR work was conducted by AFC personnel in the AFC laboratory in Colombo.

### Data collection and data management

Demographic information including age, gender, documentation of informed consent, and a history of compliance with the previously administered MDA program was collected and entered into personal digital assistants (PDA) (Dell Axim ×51, Dell Inc. Round Rock, TX or HP iPAQ 211, Hewlett Packard, Palo Alto, CA) using a preloaded survey questionnaire. Participant data, specimen ID, and test results were linked using preprinted barcode labels as described by Gass et al [21]. AFC deployed 2 or 3 teams for blood collection and 2 or 3 teams for mosquito collection in each PHI, and teams were comprised of a mixture of personnel from the district and from AFC headquarters. Data collected by multiple teams were synchronized at AFC headquarters, and data were transferred to a laptop computer using LF field office data manager software designed by the Lymphatic Filariasis Support Center, Taskforce for Global Health, Decatur, GA. Transferred files were merged to create a master database, which was backed up using an external hard drive. Specimens and laboratory test results were linked to study subject numbers (or to trap site and pool number for mosquito data) using barcodes. Deidentified, cleaned data were transferred into Excel files (Microsoft Corp., Redmond, WA) for analysis at AFC and at Washington University.

### Spatial analysis

GPS coordinates for human and mosquito sampling sites were plotted using ArcGIS 10.2.1 (ESRI, Redlands, CA) to show the location of households surveyed and mosquito trapping sites for each PHI. Waypoints were color coded to show the infection status of household residents and mosquitoes from these collection sites.

### School-based Transmission Assessment Surveys (TAS)

TAS were performed in all 8 endemic districts in late 2012 or early 2013 according to WHO guidelines. The TAS program used districts as evaluation units (EUs) in 5 cases. However, 3 districts or areas with large populations (Colombo district plus Colombo town, Gampaha, and Galle) were each divided into two EUs for TAS. All EUs met criteria for conducting TAS by having completed 5 rounds of MDA in 2006 with high MDA compliance rates (>80%). All sentinel and spot check sites in each district had Mf prevalence rates well below 1% for several years prior to TAS. Since Sri Lanka has high primary school attendance rates (>95%), TAS surveys used the cluster method to sample students in 30–35 randomly selected schools per EU[15]. Systematic selection of school children was performed with Survey Sample Builder software, SSB.V.2.1 (http://www.ntdsupport.org/resources/transmission-assessment-survey-sample-builder).

The TAS sampling strategy required filarial antigen testing of approximately 1500 primary grade children in each EU. Blood samples were collected with One Touch Ultra Soft lancet holders with disposable lancets (LifeScan, Inc., Milpitas, CA). Finger prick blood was collected into capillary tubes provided with the BinaxNow Filariasis cards, and 100 µl of blood was added directly to sample application pads of the cards according to the manufacturer's instructions. Tests were performed in the school auditorium, library, or health screening station immediately after blood collection, and read at 10 minutes. Antigen test results (positive or negative) were recorded manually using preprinted data collection forms. Children with positive filarial antigen tests were tested for microfilaremia with night blood smears as described above.

### Data analysis

We used the software program PASW Statistics 18 (SPSS, now IBM Corporation, Armonk, NY) and JMP (SAS, Cary, NC). The Chi-square test was used to assess the significance of differences in categorical variables such as antigenemia rates. The correlation between human and mosquito infection parameters was analyzed by the Spearman rank test. Logistic regression was used to assess the independence of risk factors for filarial antigenemia. Graphs were produced with GraphPad Prism V. software (La Jolla, CA). Filarial DNA rates (maximum likelihood estimates with 95% confidence intervals) were calculated with PoolScreen 2.02 [22], [23]. To sharpen the analysis of risk factors for filarial infection, we limited the analysis to 14 PHI areas where one or more people had positive filarial antigen tests. All analyses were performed assuming simple random sampling for simplicity of exposition. A generalized linear mixed model was used to estimate design effects of household-based cluster sampling used in community surveys. This analysis was performed with data from the two PHIs with the highest surveyed CFA rates.

### Ethical review

The study protocol for comprehensive surveillance in PHIs was reviewed and approved by institutional review boards at Washington University School of Medicine and at the University of Kelaniya in Sri Lanka (FWA 00013225). Prior to school surveys (both PHI surveys and TAS), study personnel held preliminary meetings with school principals and officials from the Sri Lankan Ministry of Education about the goals and procedures for the study. They also met with parents or guardians to discuss the study design and the significance of the study.

Printed participant information sheets and written consent forms were provided to participants (or to parents/guardians) in Sinhalese, Tamil and English. Written consent was obtained from adults. Participation of minors required written consent from at least one parent or guardian plus assent by the child/minor. Consent was also documented electronically into PDAs by study personnel prior to collection of health information or blood samples. TAS surveys used preprinted paper forms for parental consent and other forms for data collection (school name, child name, age, sex, and CFA result).

## Results

### Community survey results

**Figure 1:**
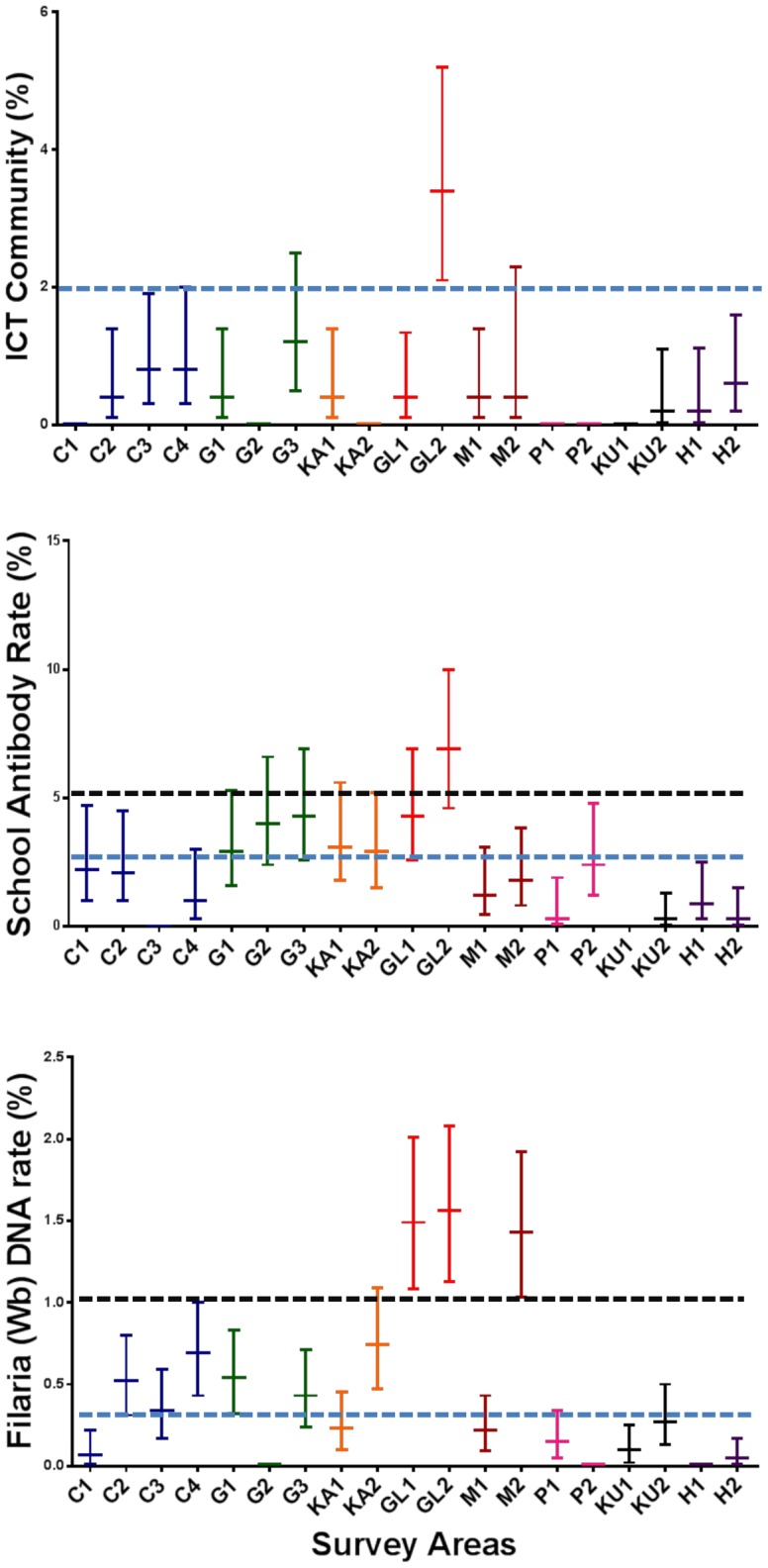
Graphic summary of comprehensive filariasis surveillance data for Public Health Inspector areas in Sri Lanka. Data shown are rates (% with 95% confidence limits as vertical lines). The dotted line in the top panel and the lower dotted lines in the two lower panels show the old provisional targets for interruption of transmission. The upper dotted lines in the two lower panels are recommended revised targets for the upper confidence limits for antibody rates in first and second grade primary school children and for filarial DNA rates in mosquitoes, respectively.

Nineteen PHI surveys were conducted in 8 districts and in Colombo town between March 2011 and July 2013. Demographic information for survey participants is provided in Table 1, and results are summarized in Table 2 and Figure 1. Community CFA rates were <2% in 17 of 19 PHIs, but upper confidence limits for CFA were >2% in 5 of 19 PHIs. Microfilaremia rates were <1% in all PHI areas studied. Sixteen of 65 CFA-positive subjects (age range 23–70 yr) were positive for Mf (mean count 14 per 60 µl range 1–51), and 68% of Mf carriers were males. The Unawatuna PHI area in Galle district had the highest rates for several filariasis parameters (Table 2 and Figure 1).

**Table 1:** Background information for Public Health Inspector (PHI) areas selected for comprehensive filariasis surveillance and demographic information for subjects enrolled in community studies conducted in these areas.

District (IU)	Population Size	PHI	PHI code	Area (km^2^)	Population size	Number of PHM areas	Age (mean)	Age (IQR)	Percent Male
Colombo	2,318,366	Katukurunda	C1	3	31,280	10	34	18–39	42.0
		Sedawatta	C2	0.6	35,680	6	34	16–46	44.0
		Mattakkuliyaa^a^	C3	4	98,091	8	40	26–52	38.8
		Borella^a^	C4	4.5	137,423	6	39	25–52	47.5
Gampaha	2, 325,675	Kelaniya	G1	24.5	23,200	6	37	17–47	39.9
		Wattala	G2	0.93	20,931	5	39	20–57	39.5
		Peliyagoda W^b^	G3	1.59	10,560	-	35	16–42	39.4
Kalutara	1,237,676	Panadura	KA1	4.5	11,200	4	42	19–52	41.8
		Kalutara N	KA2	1.6	11,728	3	39	19–50	35.8
Galle	1,066,938	Ambalangoda	GL1	6.5	13,373	5	38	18–45	44.8
		Unawatuna	GL2	11	16,636	7	38	20–48	44.3
Matara	815,625	Devinuwara	M1	6.2	15,947	4	34	18–38	40.7
		Weligama	M2	4.5	10,521	3	35	18–47	40.8
Puttalam	766,469	Chila town	P1	6.4	23,554	5	35	21–47	43.9
		Lunuwila	P2	13	24,977	4	35	21–50	42.9
Kurunegala	1,629,958	Bamunuwala	KU1	24.4	16,865	4	34	19–50	42.5
		Narammala	KU2	31	22,299	7	37	24–51	40.8
Hambantota	607,404	HT town	H1	9.2	11,521	2	36	22–52	38.3
		Tangalle	H2	1.6	10,973	3	36	21–52	40.6

a Sentinel sites (PHI) C3 and C4 were in the city of Colombo.

b Sentinel site G3 is a Public Health Field Officer area (PHFO).

**Table 2:** Summary of filariasis parameters from community (Comm) and school surveys conducted in public health inspector (PHI) areas.

District	PHI	PHI code	% MDA^a^	Mf Comm b	CFA Comm b	CFA School b	AbSchool b
Colombo	Katukurunda	C1	74.2	0	0	0	*2.2 (1.0–4.7)*
	Sedawatta	C2	81.2	0.2 (0.03–1.0)	0.4 (0.1–1.4)	0	*2.1 (0.97–4.5)*
	Mattakkuliya c	C3	29.6	0.2 (0.03–1.1)	0.8 (0.3–2.0)	0.3 (0.05–1.7)	0
	Borella c	C4	45.2	0.2 (0.04–1.1)	0.8 (0.3–2.1)	0	1.0 (0.3–3.0)
Gampaha	Kelaniy*a*	G1	66.2	0	0.4 (0.1–1.5)	0	*2.9 (1.6–5.3)*
	Wattala	G2	69.7	0	0	0	**4.0 (2.4–6.6)**
	PeliyagodaW	G3	71.0	0.4 (0.11–1.4)	1.2 (0.5–2.6)	0.3 (0.05–1.5)	**4.3 (2.6–6.9)**
Kalutara	Panadura	KA1	73.2	0	1.0 (0.4–2.3)	0	**3.1 (1.7–5.6)**
	Kalutara N	KA2	76.4	0.4 (0.11–1.4)	2.0 (1.1–3.6)	0.5 (0.15–1.9)	*2.9 (1.5–5.2)*
Galle	Ambalangoda	GL1	29.9	0	0.4 (0.1–1.3)	0.5 (0.14–1.8)	**4.3 (2.6–6.9)**
	Unawatuna	GL2	25.3	*0.9 (0.40–2.2)*	**3.4 (2.1–5.2)**	*0.8 (0.28–2.4)*	**6.9 (4.6–10)**
Matara	Devinuwara	M1	80.5	0	0.4 (0.1–1.4)	0	1.2 (0.48–3.1)
	Weligama	M2	85.5	0.6 (0.20–1.7)	1.0 (0.4–2.3)	0.6 (0.16–2.0)	1.8 (0.82–3.8)
Puttalam	Chila town	P1	82.1	0.2 (0.04–1.1)	0	0	0.3 (0.1–1.9)
	Lunuwila	P2	78.9	0	0	0	*2.4 (1.2–4.8)*
Kurunegala	Bamunuwala	KU1	89.7	0	0	0	0
	Narammala	KU2	88.3	0.2 (0.03–1.1)	0.2 (0.03–1.1)	0	0.3 (0.05–1.3)
Hambantota	HT town	H1	78.5	0	0.2 (0.03–1.1)	0	0.9 (0.29–2.5)
	Tangalle	H2	83.4	0	0.6 (0.20–1.7)	0	0.3 (0.05–1.5)

a Surveyed rates for ingestion of antifilarial medications during the national mass drug administration (MDA) program 2002–06.

b Prevalence rates are mean values (95% CI) by PHI. Results are shown as pass (regular font), borderline (*italics*) or fail (**bold**) based on provisional endpoint criteria described in the Introduction.

c Study sites C3 and C4 were in the city of Colombo.

CFA rates were higher in males than females when data from all community surveys were considered (1.01% vs. 0.42%, *P*<0.001) and when localities with no positive CFA tests were excluded from the analysis (1.39% vs. 0.57%, *P*<0.001) (Table 3). CFA rates were also higher in adults than in children, and this was especially true for people older than 30 years (Table 3). CFA rates were lower in people who reported having used a bed net the night before their interview (all localities), but the difference was not statistically significant (0.57% vs. 0.92%, *P* = 0.06). However, the reduced CFA rate in bed net users was significant when localities with no positive CFA tests were excluded from the analysis (0.76% vs. 1.29%, *P* = 0.04). Bed net users also had lower rates of microfilaremia in these localities (0.17% vs. 0. 52%, *P* = 0.012).

**Table 3:** Filariasis infection parameters by age and gender in Public Health Inspector^a^ areas.

Age Range (Yr)	Males	CFA^b^ % (CI)	Females	CFA^b^ % (CI)	Total (%, CI)
10–15	1/462	0.22 (0.04–1.22)	1/418	0.24 (0.04–1.34)	2/880 (0.23, 0.06–0.82)
16–20	2/352	0.57 (0.16–2.05)	0/365	0	2/717 (0.28, 0.08–1.01)
21–30	4/447	0.90 (0.35–2.28)	3/692	0.43 (0.15–1.27)	7/1139 (0.62, 0.30–1.26)
31–40	11/490	2.25 (1.26–3.97)	4/838	0.48 (0.19–1.22)	15/1328 1.13, 0.69–1.86)
41–50	12/487	2.46 (1.42–4.26)	3/758	0.40 (0.13–1.16)	15/1245 (1.2, 0.73–1.98)
51–60	8/395	2.03 (1.03–3.95)	4/633	0.63 (0.25–1.61)	12/1028 (1.17, 0.67–2.03)
≥61	3/326	0.92 (0.31–2.67)	9/493	1.83 (0.96–3.43)	12/819 (1.47, 0.84–2.54)

a Circulating filarial antigen (CFA) results from 14 public health inspector areas (PHIs) with one or more CFA positives were included in this analysis.

b Data shown are CFA prevalence rates (95% CI).

Reported compliance rates for ingestion of antifilarial medications during the national MDA program were high in most PHIs surveyed, but very low rates were reported in PHIs in Galle district and in Colombo town (Table 2). These results are consistent with low surveyed compliance rates previously reported for these areas [10]. CFA rates in community surveys were significantly lower in people who reported that they had ingested antifilarial medication during the national MDA program (0.45% vs. 1.15%, *P* = 0.001).

Logistic regression was used to assess the independence of different risk factors for CFA for all surveyed communities and for the subset of communities with one or more subjects positive for CFA (Table 4). Gender, age, and prior MDA treatment were significant independent indicators of risk, but reported bed net use was not.

**Table 4:** Multivariable logistic regression of risk factors for filarial antigenemia in community survey data.

	All PHI areas^a^		Infected Areas^b^ Only	
Factor	Odds Ratio (95% CI)	*P*	Odds Ratio (95% CI)	*P*
Male gender	**2.48** (1.51–4.19)	0.0003	**2.54** (1.54–4.29)	0.0002
Denied any prior intake of antifilarial medication	**2.55** (1.55–4.22)	0.0002	**2.14** (1.30–3.54)	0.003
Denied use of bed net the night before the survey	**1.34** (0.80–2.21)	0.25	**1.45** (0.87–2.39)	0.15
Age (per decade)	**1.32** (1.15–1.52)	.0001	**1.31** (1.14–1.51)	0.0002

a Results from all 19 public health inspector (PHI) areas that were surveyed.

b This analysis was restricted to results from 14 PHI areas where one or more persons tested had a positive filarial antigen test.

Intraclass correlations by household in the two locations with the highest filarial infection rates were 0.16 and 0.08, and these values correspond to design effects of 1.6 and 1.3.

### School survey results

CFA rates were very low in children tested in school surveys, and this was consistent with TAS results presented below. Anti-filarial antibodies were detected in primary school children in 17 of 19 PHIs. Antibody rates exceeded the target rate of 2% in 10 of 19 PHIs; five PHIs had borderline elevated antibody rates, and 5 others had higher rates with upper confidence limits >5%. Only three of 137 children with positive antibody tests (out of 6198 children tested for antibody from all 19 PHI areas) had positive CFA tests, and all three of these children were Mf negative.

### Antifilarial antibodies in community surveys

Community antibody testing was performed in a subset of PHIs that were surveyed in the comprehensive surveillance study (Table S1). Although CFA and Mf rates in these communities were below provisional target levels, community antibody rates were high in all of these PHIs, and this probably reflects high infection rates that were present in these areas prior to implementation of the national MDA program.

### Relationships between different human filariasis parameters in community and school surveys

Human filariasis parameters tended to be significantly correlated with each other [e.g., community Mf rate vs. community CFA rate (r = 0.63, *P = *0.0018), school CFA rate vs. school antibody rate (r = 0.5, *P = *0.0142), and community CFA rate vs. school CFA rate (r = 0.69; *P = *0.0006)].

### Transmission assessment survey results

More than 17,000 primary grade school children were tested in TAS in 337 schools located in 11 EUs in 8 districts and in Colombo town (Table 5). The numbers of positive CFA tests were well below the TAS threshold level of 18 (critical cut-off value) in all EUs. Thus all EUs “passed” TAS including the coastal Galle District EU, where high rates for filariasis markers were noted in two PHI study areas. None of the 16 children with positive CFA tests in TAS surveys had microfilaremia. All CFA-positive children were treated with anti-filarial medications and follow-up surveys are in progress or planned to further assess people in areas with positive children.

**Table 5:** Transmission assessment survey (TAS^a^) results from 11 evaluation units (EUs) in 8 districts^b^ in in Sri Lanka.

Evaluation Unit	Population size/EU	Number of primary grade schools included	Number of primary grade children tested	Number of children positive for filarial antigenemia^c^
Colombo-RDHS	1,761,010	30	1716	2 (0.12, 0.03–0.4)
Colombo-city	557,356	30	1555	2 (0.13, 0.04–0.4)
Gampaha I	898,731	30	1642	1 (0.06, 0.01–0.3)
Gampaha II	1,426,944	30	1462	0 (0)
Kalutara	1,237,676	30	1585	4 (0.3, 0.10–0.6)
Galle I	719,911	31	1557	7 (0.45, 0.22–0.9)
Galle II	347,027	31	1543	0 (0)
Matara	815,625	30	1591	0 (0)
Puttalam	766,469	30	1583	0 (0)
Kurunegala	1,629,958	35	1692	0 (0)
Hambantota	607,404	30	1553	0 (0)
Total	10,768,112	337	17479	16 (0.1, 0.06–0.1)

a The critical cutoff value for assessing interruption of transmission was 18 in all EUs.

b The 8 endemic districts were MDA implementation units.

c BinaxNOW Filariasis tests were used for detection of filarial antigenemia. Data shown are the number of positive tests (% positive and 95% CI).

### Filarial DNA rates in mosquitoes

Almost 3,900 pools (20 mosquitoes per pool) of blood fed, gravid or semi-gravid mosquitoes collected in 19 PHI areas were tested for filarial DNA by qPCR (Table 6). Filarial DNA rates exceeded the target of 0.25% in 10 of 19 PHIs. Mosquitoes from both PHIs surveyed in Galle district and one in Matara district had parasite DNA rates of more than 1%, and these rates were comparable to those seen in some filariasis endemic areas in Egypt with continued filariasis transmission following one or two rounds of MDA [24]. Upper confidence limits for filarial DNA rates were ≥1% in 5 of 19 PHIs surveyed. On the other hand, three of 19 PHIs surveyed had no positive mosquito pools. Most of the other filariasis parameters were also low in these PHIs. Mosquito DNA samples from Wattala were retested by qPCR at Washington University and confirmed to be negative.

**Table 6:** Filarial DNA rates in Sri Lankan *Culex quinquefasciatus* mosquitoes by Public Health Inspector area.

District	PHI area^a^	PHI code	Number of mosquitoes tested	Number of pools tested b	Number (%) of positive pools	Filarial DNA rates in mosquitoes c
Colombo	Katukurunda	C1	4000	200	3 (1.5)	0.07 (0.01–0.22)
	Sedawatta	C2	4480	224	21 (9)	**0.52 (0.31–0.80)**
	Mattakkuliya	C3	4000	200	13 (6.5)	*0.34 (0.17–0.59)*
	Borella	C4	4000	200	26 (13)	**0.69 (0.43–1.0)**
Gampaha	Kelaniya	G1	4320	216	22 (10)	**0.54 (0.32–0.83)**
	Wattala	G2	4000	200	0 (0)	0
	PeliyagodaW	G3	4080	203	17 (8)	*0.43 (0.24–0.71)*
Kalutara	Panadura	KA1	4000	200	9 (4.5)	0.23 (0.10–0.45)
	Kalutara N	KA2	4080	204	28 (14)	**0.74 (0.47–1.09)**
Galle	Ambalangoda	GL1	4000	200	52 (26)	**1.49 (1.08–2.01)**
	Unawatuna	GL2	4000	200	54 (27)	**1.56 (1.13–2.08)**
Matara	Devinuwara	M1	4160	208	9 (4)	0.22 (0.09–0.43)
	Weligama	M2	4080	204	51 (25)	**1.43 (1.03–1.92)**
Puttalam	Chila town	P1	4000	200	6 (3)	0.15 (0.05–0.34)
	Lunuwila	P2	4160	208	0 (0)	0
Kurunegala	Bamunawala	KU1	4160	208	4 (1.9)	0.10 (0.02–0.25)
	Narammala	KU2	4160	208	11 (5.2)	*0.27 (0.13–0.50)*
Hambantota	HT town	H1	4000	200	0 (0)	0
	Tanagalle	H2	4080	204	2 (1)	0.05 (0.01–0.15)

a Sentinel sites (PHIs) C3 and C4 were located in the city of Colombo. Sentinel site G3 is a PHFO area.

b Each pool included 20 mosquitoes (blood fed, gravid and semigravid).

c Filarial DNA was detected by qPCR. Rates of filarial DNA in mosquitoes (maximum likelihood and 95% CI) were estimated using PoolScreen2. Results are shown as pass (regular font), borderline (*italics*) or fail (**bold**) based on provisional endpoint criteria described in the Introduction.

The percentages of positive mosquito trap sites were highly variable in different PHIs, and these rates were strongly correlated with percentages of pools positive for filarial DNA (r = 0.99, *P*<0.0001), community CFA rates (r = 0.72, *P = *0.0003), and school CFA rates (r = 0.77; P<0.0001). Percentages of mosquito pools positive for filarial DNA were highly correlated with community CFA rates (r = 0.71, *P = *0.0001) and school CFA rates (r = 0.79, P<0.0001). In addition, percentages of houses with at least one CFA positive resident were highly correlated with percentages of mosquito trap sites with filarial DNA in mosquitoes (r = 0.75, *P = *0.0001) (Table S2) and with percentages of mosquito pools that contained filarial DNA (r = 0.73; *P = *0.0002).

### Spatial analysis of filarial infections in humans and mosquitoes

**Figure 2:**
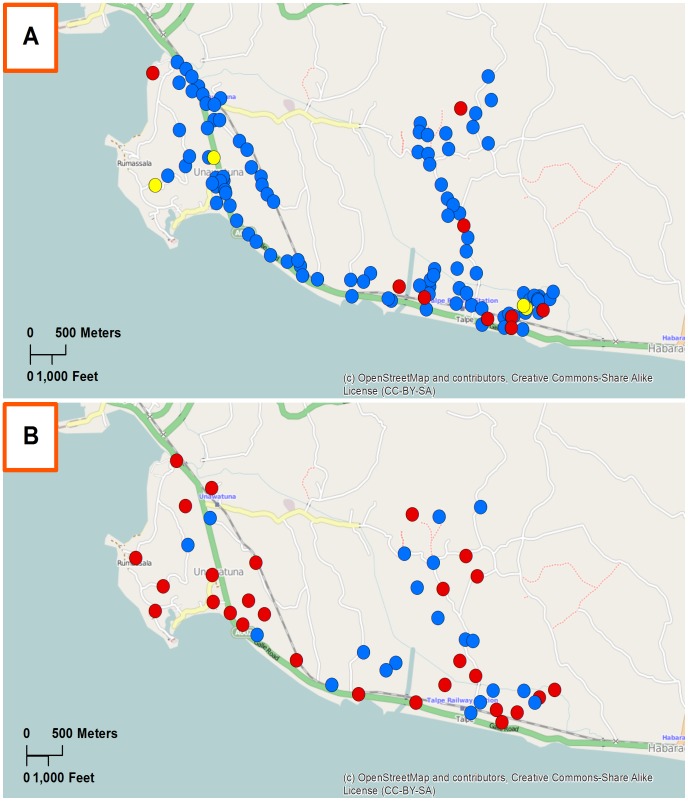
Distribution of households and mosquito collection sites tested for filariasis in Unawatuna PHI area in Galle district. Panel A. Blue waypoints indicate households (HH) where all tested residents had negative filarial antigen tests; waypoints in red (CFA positivity) or yellow (microfilaremia and CFA positivity) indicate houses with at least one infected subject. Panel B shows molecular xenomonitoring results. Trap sites with no mosquito pools positive for filarial DNA are shown in blue, and traps with one or more positive mosquito pools are shown in red. Filarial DNA was detected in mosquitoes collected in 60% of the traps in this PHI.

GPS data for PHI areas with high and low rates of persistent LF are shown in Figures 2 and S1. These maps show that sampled households and mosquito collection sites were nicely dispersed to cover the study areas. Infections in human and parasite DNA in mosquitoes tended to be dispersed in most study areas.

### Longitudinal results from Peliyagodawatta

A pilot LF surveillance study was performed in 2008 in Peliyagodawatta, which is located in Gampaha district just outside of the city of Colombo. The area was resurveyed in 2011, approximately 2.5 years after the baseline study. This is a low income, peri-urban area with high mosquito densities, and no intervention for LF control was undertaken in this area between 2008 and 2011. Results from the two surveys are summarized in Table 7. Several filariasis parameters were lower in 2011 than in 2008. While only the reduction in community CFA was statistically significant, the trend toward reduction was present for all of these parameters apart from Mf rate, which was already very low in 2008.

**Table 7:** Comparison of filarial infection parameters in Peliyagodawatta^a^ in 2008 and 2011.

Filarial infection markers	No. tested 2008	Prevalence b 2008	No. tested 2011	Prevalence b 2011	*P* value c
Mf Community d	944	0.4 (0.16–1.08)	5	0.4 (0.1–1.4)	0.73
CFA Community d	945	3.8 (2.76–5.23)	504	1.2 (0.5–2.4)	0.01
CFA age 6–8	265	1.9 (0.81–4.34)	377	0.3 (0.05–1.49)	0.09
Filarial DNA rate in mosquitoes	277 pools	0.75 (0.52–1.06)	203 pools	0.43 (0.24–0.7)	NS
Number (%) of mosquito pools positive for filarial DNA		39/277 (14%)		17/203 (8.3%)	0.07

a Peliyagodawatta is a Public Health Field Officer area in Gampaha district.

b Results shown are % positive (95% CI). Filarial DNA rates shown are maximum likelihood estimates (with 95% CI).

c *P* values are based on χ^2^. NS, not significant.

d Community microfilaremia (Mf) and circulating filarial antigenemia (CFA) rates are for ages ≥10 years. Mf rates are based on night blood smear results from all subjects in 2008 and from CFA positives only in 2011.

The first survey in Peliyagodawatta identified 37 amicrofilaremic subjects with positive filarial antigen tests. These people were not treated for LF at that time. Twenty-five of these people were retested in 2010, approximately 18 months after the first survey; others had moved or were otherwise not available for follow-up. Only 12 of 25 subjects were still CFA-positive (48%), and only 1 of 25 was microfilaremic by 60 µl night blood smear. None of the subjects reported symptoms or signs of clinical filariasis during the 18 month interval. All subjects with filarial antigenemia were treated in 2011.

## Discussion

This study has provided interesting data on the status of LF in Sri Lanka approximately 6 years after completion of the country's MDA program, and it has important implications for post-MDA surveillance activities in other LF-endemic countries around the world. Few countries participating in GPELF have been studied as thoroughly as Sri Lanka.

### Has Sri Lanka successfully eliminated LF?

The term “LF elimination” has been interpreted in different ways, but WHO documents clearly state that one goal of LF elimination programs is interruption of transmission [15]. WHO is also responsible for deciding when countries have eliminated LF. Pending their review, we think it is important to recognize the achievements of Sri Lanka's Anti-Filariasis Campaign, which is one of the finest LF elimination programs in the world. The program has reduced Mf rates to less than 1% in all sentinel and spot check sites, all EUs easily passed TAS criteria for stopping MDA, and the AFC has a network of clinics that provide care to thousands of lymphedema patients in all endemic districts. By these criteria, Sri Lanka has achieved several WHO targets and the country is on track to achieve elimination. If WHO determines that Sri Lanka has not met criteria for LF elimination, we believe that the organization should develop criteria and a recognition program for countries that can document this level of superb control, because this pre-elimination status is a significant achievement in public health and an important step on the road to LF elimination. External recognition of “superb control” or “near elimination” may help national programs obtain political support and resources needed for the difficult last mile required for true elimination.

### What is the relative value of different approaches and technologies for post-MDA surveillance of LF?

While protocols for transmission assessment surveys are based on solid sampling principles, the sensitivity of TAS for detecting ongoing transmission of LF has not been adequately tested in field studies [15]. Our results clearly show that TAS performed according to WHO guidelines were not sensitive for detecting ongoing LF transmission in Sri Lanka. There are a number of reasons for this. First, we believe that EUs of 1 to 2 million are too much too large, because risk factors that affect LF transmission often vary widely across such large populations/areas. This problem could be mitigated by reducing the size of EUs (for example, to areas with populations of 100,000 or less), but that would significantly increase the cost of TAS. A second problem with TAS is that filarial antigenemia rates in young children are sometimes very low in areas with ongoing LF transmission. Our study showed that CFA rates in school aged children were much lower than those in adults. Therefore, the sensitivity of TAS might be improved by using a similar cluster sampling method to test adults (for example, those attending primary health clinics) instead of children in schools. A recent report from Togo described the use of other types of passive surveillance for assessing LF following MDA [25].

Since anti-filarial antibody rates are uniformly higher than antigenemia rates in LF-endemic populations, another potential solution for the problem of low TAS sensitivity would be to substitute antibody testing for antigen testing in TAS for samples of school-aged children. Antibody results from the present study using a commercially available ELISA kit provide a proof of principle for this approach. However, ELISA testing may not be feasible for all LF programs, and available rapid-format antibody tests have not yet been validated for this purpose.

Results from this study strongly support the use of molecular xenodiagnosis for post-MDA surveillance in areas where LF is transmitted by *Culex* mosquitoes. MX does not require collection of blood samples or active participation by large numbers of people in endemic areas. However, MX does require cadres of skilled personnel, specialized laboratory facilities, and funds for consumables. While MX was performed by MOH personnel in this study, this required significant external inputs including equipment, supplies, training of personnel, and funds for mosquito collection. Also, additional work is needed to develop and validate sampling methods for assessment of mosquito DNA rates in areas larger than PHIs.

To summarize this section of the Discussion, while TAS surveys may be useful for decisions regarding stopping MDA, they are not sufficient to show that LF transmission has been interrupted. The sensitivity of TAS might be improved by reducing the size of EUs or by sampling adults instead of school-aged children. We recommend antibody testing of children using TAS sampling methods and/or MX (especially in areas believed to be at high risk) to complement antigen-test based TAS, because these methods appear to be more sensitive than TAS for detecting ongoing LF transmission.

### Revised targets for LF elimination programs

This study has provided new insight regarding provisional targets for MDA programs that were suggested in 2007 based on data from Egypt [16]. Since there is uncertainty surrounding all point estimates, we now recommend using confidence intervals to express targets as illustrated in Figure 1. The new suggested target for the antifilarial antibody rate in first and second grade school children is to have an upper confidence limit of <5%. The new target for MX (*Culex* mosquitoes) is to have an upper confidence limit of the maximum likelihood estimate of <1%. The new target for the community CFA rate (age >9) is to have an upper confidence limit of <2%. This target provides a very high level of confidence that the Mf rate will be less than 0.5% in the community with a much smaller sample size than what would be required for Mf testing. Additional studies will be needed to test the new proposed targets in different regions. We believe that these targets will be helpful for identifying areas that require continued surveillance.

### Next steps for areas that may have ongoing transmission following MDA

Existing guidelines do not adequately address this issue. Four options to consider are resumption of MDA, implementation of test and treat programs, vector control, and watchful waiting. It may be difficult to justify resumption of MDA when Mf rates are well below 1% when one considers that many of those with persistent infections may have been noncompliant with MDA in the past. Test and treat campaigns may be more efficient for finding and treating those with persistent infections than MDA, and the Sri Lanka AFC has started to do this in Galle district. Our results suggest that adult males and people who do not recall having taken MDA in the past should be considered to be high priority target groups for test and treat programs.

WHO has recommended vector control as a post MDA strategy [26]. Although vector control can be difficult to implement at the scale needed for LF elimination, surveillance results may identify hot spot areas where focused vector control may be feasible. Our finding that CFA rates were lower in people who reported using bed nets is interesting, although the logistic regression analysis suggested that lack of bed net use was not an independent risk factor for filarial infection. Bed nets are popular in Sri Lanka because of the mosquito nuisance factor and the risk of dengue. Beneficial effects of bed nets for LF have been reported from areas with *Anopheles* transmission [27], [28]. The Sri Lanka government should consider implementing a health education campaign to reinforce the popularity of bed nets and increase usage rates in areas with persistent LF.

The longitudinal data from Peliyagodawatta are intriguing, because they suggest that some areas with filariasis parameters that do not meet our provisional criteria for interruption of transmission may spontaneously improve over time. Thus the strategy of watching, waiting, and retesting may be the best course of action for some areas with persistent LF. Other data from Peliyagodawatta on the natural history of filarial antigenemia in amicrofilaremic individuals in the post-MDA setting are reassuring. These results suggest that there is no pressing need to actively identify and treat asymptomatic and amicrofilaremic persons with positive filarial antigen tests following MDA. This is because the risk of such people developing microfilaremia is low, and antigenemia often clears over time without treatment.

We believe that this study has contributed significant new information regarding post-MDA surveillance and low level persistence of filariasis following MDA. LF elimination is a dynamic process [29], and point estimates of persistent infection may be less important than trends over time. For this reason, we plan to restudy Peliyagodawatta and several other PHIs with elevated LF parameters three years after the evaluations described in this publication.

## Supporting Information

Figure S1 Distribution of households and mosquito collection sites tested for filariasis in Chila Town PHI area in Puttalam district which has less evidence of persistent filariasis than Unawatuna PHI (shown in Fig 2). Panel A. Blue waypoints indicate households (HH) where all tested residents had negative filarial antigen tests; waypoints in red indicate houses with at least one infected subject (CFA positive). Panel B shows molecular xenomonitoring results. Trap sites with no mosquito pools positive for filarial DNA are shown in blue, and traps with one or more positive mosquito pools are shown in red. Filarial DNA was detected in mosquitoes collected in 10% of the traps in this PHI area.
(TIFF)

Table S1 Community rates for circulating filarial antigenemia (CFA), microfilaremia (Mf), and IgG4 antibodies to filarial antigen Bm14 in selected public health inspector.
(DOCX)

Table S2 Filarial infections by household and mosquito trap site in different Public Health Inspector (PHI) areas in Sri Lanka.
(DOCX)

Checklist S1 STROBE statement. Checklist of items included in this cross-sectional study Rao et al., A Comprehensive Assessment of Persistent Lymphatic Filariasis in Sri Lanka Six Years after Cessation of Mass Drug Administration.
(DOC)
