# Correction: A Comprehensive Assessment of Lymphatic Filariasis in Sri Lanka Six Years after Cessation of Mass Drug Administration

**DOI:** 10.1371/journal.pntd.0003428

**Published:** 2014-12-12

**Authors:** The PLOS Neglected Tropical Diseases Staff

There is an error in reference 5. The correct reference is: AntifilariasisCampaign (2013) Annual reports. Ministry of Health, Sri Lanka Available at http://www.filariasiscampaign.health.gov.lk/
