# Supplementary figures and images for: A Comprehensive Assessment of Lymphatic Filariasis in Sri Lanka Six Years after Cessation of Mass Drug Administration
Source: PLoS Negl Trop Dis. 2014 Nov 13;8(11):e3281. doi: 10.1371/journal.pntd.0003281 (PMC4230885; doi:10.1371/journal.pntd.0003281)

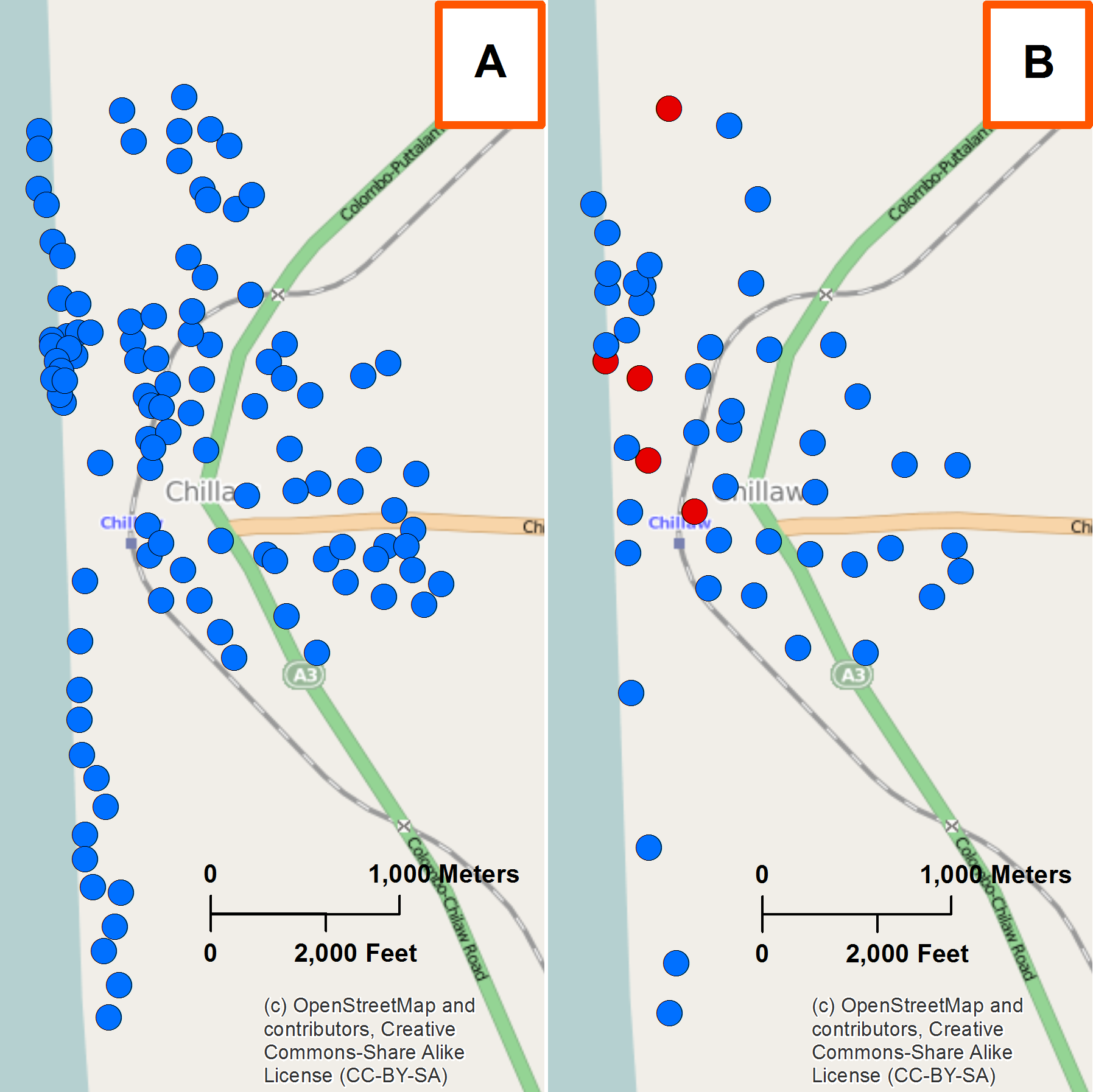

Supplement: Figure S1 — Distribution of households and mosquito collection sites tested for filariasis in Chila Town PHI area in Puttalam district which has less evidence of persistent filariasis than Unawatuna PHI (shown in Fig 2). Panel A. Blue waypoints indicate households (HH) where all tested residents had negative filarial antigen tests; waypoints in red indicate houses with at least one infected subject (CFA positive). Panel B shows molecular xenomonitoring results. Trap sites with no mosquito pools positive for filarial DNA are shown in blue, and traps with one or more positive mosquito pools are shown in red. Filarial DNA was detected in mosquitoes collected in 10% of the traps in this PHI area. (TIFF) [file pntd.0003281.s001.tiff]
