# Supplementary material for: A Comprehensive Assessment of Lymphatic Filariasis in Sri Lanka Six Years after Cessation of Mass Drug Administration
Source: PLoS Negl Trop Dis. 2014 Nov 13;8(11):e3281. doi: 10.1371/journal.pntd.0003281 (PMC4230885; doi:10.1371/journal.pntd.0003281)
Supplement: Table S1 — Community rates for circulating filarial antigenemia (CFA), microfilaremia (Mf), and IgG4 antibodies to filarial antigen Bm14 in selected public health inspector. (DOCX) [file pntd.0003281.s002.docx]

Table S1. Community rates for circulating filarial antigenemia (CFA), microfilaremia

(Mf), and IgG4 antibodies to filarial antigen Bm14 in selected public health inspector

(PHI) areas.

| **District** | **PHI** | **CFA (%)** | **Mf (%)** **^a^** | **Antibodies to Bm14** ^b^ |
| --- | --- | --- | --- | --- |
| Colombo | Katukurunda | 0/528 (0) | 0 (0) | 19/244 (7.8, 5-12%) |
|  | Sedawatta | 2/515 (0.4) | 1 (0.1) | 59/498 (11.8, 9-14%) |
| Gampaha | Kelaniya | 2/503 (0.4) | 0 (0) | 90/413 (21.8, 18-26%) |
| Puttalam | Chila town | 0/507 (0) | 1/487 (0.2) | 21/473 (4.4, 2.9-6.7%) |
|  | Lunuwila | 0/508 (0) | 0/493 (0) | 34/482 (7.1, 5.0-9.7%) |

^a^Mf testing was performed for all subjects studied in Puttalam district. Mf testing was

only performed for persons with positive CFA tests in Colombo and Gampaha districts.

^b^Results shown in parentheses are % positive with 95% CI.
