# Supplementary material for: A Comprehensive Assessment of Lymphatic Filariasis in Sri Lanka Six Years after Cessation of Mass Drug Administration
Source: PLoS Negl Trop Dis. 2014 Nov 13;8(11):e3281. doi: 10.1371/journal.pntd.0003281 (PMC4230885; doi:10.1371/journal.pntd.0003281)
Supplement: Table S2 — Filarial infections by household and mosquito trap site in different Public Health Inspector (PHI) areas in Sri Lanka. (DOCX) [file pntd.0003281.s003.docx]

Table S2. Filarial infections^a^ by household and mosquito trap site in different Public

Health Inspector (PHI) areas in Sri Lanka.

| **District** | **PHI** | **Number (%) of CFA positive houses** | **Number (%) of positive mosquito trap sites** |
| --- | --- | --- | --- |
| Colombo | Katukurunda | 0/150 (0) | 3 (5%) |
|  | Sedawatta | 1/113 (0.9) | 16 (29%) |
|  | Mattakkuliya | 4/104 (3.8) | 11 (21.6%) |
|  | Borella | 4/103 (3.8) | 21 (35%) |
| Gampaha | Kelaniya | 2/115 (1.7) | 16 (40%) |
|  | Wattala | 0/121 (0) | 0 (0%) |
|  | Peliyagoda W | 5/114 (4.4) | 14 (23%) |
| Kalutara | Panadura | 4/138 (2.9) | 8 (15%) |
|  | Kalutara N | 8/120 (6.6) | 22 (43%) |
| Galle | Ambalangoda | 2/135 (1.5) | 29 (57%) |
|  | Unawatuna | 15/116 (13) | 30 (60%) |
| Matara | Devinuwara | 2/123 (1.6) | 7 (13%) |
|  | Weligama | 5/109 (4.6) | 31 (53%) |
| Puttalam | Chila town | 1/111 (0.9) | 5 (10%) |
|  | Lunuwila | 0/102 (0) | 0 (0%) |
| Kurunegala | Bamunuwala | 0/116 (0) | 4 (8%) |
|  | Narammala | 1/106 (0.9) | 7 (12%) |
| Hambantota | HT town | 1/109 (0.9) | 0 (0%) |
|  | Tanagalle | 3/112 (2.7) | 2 (3.5%) |

^a^ Filarial infections in humans were detected by testing for circulating filarial antigenemia (CFA). Houses with one or more infected residents were considered to be infected houses. Filarial DNA in mosquitoes collected in gravid traps was detected by qPCR. Percentages of houses with human infections and percentages of gravid traps with infected mosquitoes by PHI were strongly correlated (r = 0.75, *P =* 0.0001).
